# Engineering of Nanostructured WO_3_ Powders for Asymmetric Supercapacitors

**DOI:** 10.3390/nano12234168

**Published:** 2022-11-24

**Authors:** Giacometta Mineo, Mario Scuderi, Gianni Pezzotti Escobar, Salvo Mirabella, Elena Bruno

**Affiliations:** 1 Physics and Astronomy Department “Ettore Majorana”, University of Study of Catania, via S. Sofia 64, 95123 Catania, Italy; 2 CNR—Institute for Microsystems and Microelectronics, Catania University Unit, via S. Sofia 64, 95123 Catania, Italy; 3 CNR—Institute for Microsystems and Microelectronics, Strada VIII 5, 95121 Catania, Italy

**Keywords:** tungsten trioxide, nanostructures, energy storage, electrochemistry, asymmetric supercapacitors

## Abstract

Transition metal oxide nanostructures are promising materials for energy storage devices, exploiting electrochemical reactions at nanometer solid–liquid interface. Herein, WO_3_ nanorods and hierarchical urchin-like nanostructures were obtained by hydrothermal method and calcination processes. The morphology and crystal phase of WO_3_ nanostructures were investigated by scanning and transmission electron microscopy (SEM and TEM) and X-ray diffraction (XRD), while energy storage performances of WO_3_ nanostructures-based electrodes were evaluated by cyclic voltammetry (CV) and galvanostatic charge–discharge (GCD) tests. Promising values of specific capacitance (632 F/g at 5 mV/s and 466 F/g at 0.5 A/g) are obtained when pure hexagonal crystal phase WO_3_ hierarchical urchin-like nanostructures are used. A detailed modeling is given of surface and diffusion-controlled mechanisms in the energy storage process. An asymmetric supercapacitor has also been realized by using WO_3_ urchin-like nanostructures and a graphene paper electrode, revealing the highest energy density (90 W × h/kg) at a power density of 90 W × kg^−1^ and the highest power density (9000 W/kg) at an energy density of 18 W × h/kg. The presented correlation among physical features and electrochemical performances of WO_3_ nanostructures provides a solid base for further developing energy storage devices based on transition metal oxides.

## 1. Introduction

The finite supply of fossil fuels, climate change, and the growing world energy demand inspire great interest in the scientific community in developing renewable and sustainable energy resources as well as efficient energy storage technologies [1,2]. In the panorama of energy storage systems, electrical double-layer capacitors (EDLC) represent a promising solution in high-power density applications, since the electrochemical reactions driving the energy storage mechanism occur at the surface of active material [3]. On the other hand, these surface mechanisms result in a low energy density, since only a limited region of the active material is used [3]. Batteries, contrariwise, are the choice when high energy density is required since the energy storage mechanism is due to redox reactions involving almost all active material [1,4,5]. To overcome the differences between EDLC and batteries, a new device, called a pseudocapacitor, has been developed. The charge storage mechanism of a pseudocapacitor is based on fast faradic reactions occurring at or near the electrode surface and leading to high levels of charge storage [6,7]. Indeed, a pseudocapacitor works as a battery, but redox reactions occur at the surface, like in an EDLC [8,9], thus joining the intermediate power and energy density of both kinds of devices. This behavior results in a combination of diffusion and surface-limited charge storage mechanisms, which can be individuated by cyclic voltammetry analysis [10]. Pseudocapacitors are typically obtained by using highly porous transition metal oxide-based electrodes [8,9] and carbon-based materials. Di Mari et al. [11] synthesized ZnO nanostars which exhibit a specific capacity of 94 F/g at 5 mV/s without any substrate contributions. Nazir et al. [12] synthesized porous carbonaceous materials from banana peel waste and decorated them with nitrogen and sulfur and the obtained powder exhibited a specific capacitance of 220 F/g at 0.5 A/g. Among metal oxide semiconductors, WO_3_ has emerged as favorable material thanks to its properties. Indeed, WO_3_ is an *n*-type semiconductor with high electrochemical stability in acidic environments, high theoretical electronic conductivity (10–10^−6^ s × cm^−1^), high intrinsic density (>7 g × cm^−3^), and high specific capacitance (*C_s_*) [13].

Several WO_3_ nanostructures for energy storage applications can be found in the literature, with a very ample range of *C_s_* [14,15,16,17]. The low-cost and simple hydrothermal method can produce hexagonal WO_3_ nanorods [18] with *C_s_* of 538 F/g at 5 mV/s, hexagonal WO_3_ nanofibers [19] with *C_s_* of 436 F/g at 1 A/g, hexagonal WO_3_ nanostructures [20] with *C_s_* of 377 F/g at 2 mV/s. A careful and reproducible determination of specific capacitance is compulsory, linked to a detailed morphological and crystallographic characterization, to better understand the ultimate performance for energy storage of this material. Among the effective strategies to further improve the electrochemical properties of WO_3_, crystal phase modulation can be effective, since the hexagonal crystalline structure of WO_3_ is widely claimed in the literature as the most suitable for electrochemical charge storage due to favorable H^+^ intercalation-release pathways, with respect to the monoclinic counterpart which shows very low specific capacitance (240 F/g at 5 mV/s) [20].

Here, we report a detailed structural and electrochemical characterization of hexagonal WO_3_ nanostructures synthesized by the hydrothermal method. A careful protocol for electrode realization and evaluation of charge storage and release is described with modeling of the storage process in terms of surface and diffusion-controlled mechanisms. At last, an asymmetric supercapacitor (ASC) is realized by using a WO_3_ nanostructures electrode as an anode and graphene paper (GP) electrode as a cathode.

## 2. Materials and Methods

### 2.1. Synthesis of WO_3_ Nanorods

Hexagonal WO_3_ nanorods are synthesized as reported in previous work [21] by hydrothermal synthesis. Briefly, 0.825 g of Na_2_WO_4_•2H_2_O and 19 mL of deionized water were mixed until a clear solution is obtained. A certain amount of 3 M HCl is added to reach a 2.2 pH. The obtained precursor solution is transferred into a 25 mL stainless steel autoclave and 0.5% mg of NaCl is added as capping agent. The thermal treatment is conducted at 180 °C for 3 h. The autoclave is cooled down naturally. A WO_3_ powder is obtained by washing the synthetized nanoparticles with ethanol and water several times, followed by centrifugation (6000 rpm for 10 min), and final drying onto a hot plate for 1 h in air at 50 °C (denoted as WO_3__AsPrep). Some samples underwent a calcination process on a hot plate at 70 °C for 40 min or at 400 °C for 60 min (denoted as WO_3__Ann70 or WO_3__Ann400, respectively).

### 2.2. Preparation of Electrodes

The obtained WO_3_-based nanostructures were used for the realization of pastes (20 mg of nanostructures powder, 0.1 mL of Nafion, and 4 mL of deionized water). Part of these pastes was filtered by a 1.2 μm filter. Graphene paper (GP, 2 × 1 cm^2^, 240 μm thick, Sigma Aldrich, St. Louis, MO, USA,) was used as substrates and the electrodes are produced by drop casting the obtained WO_3_-based pastes. The acronyms “NF” and “F” are used for electrodes prepared with the non-filtered and filtered WO_3_-based pastes, respectively. A Mettler Toledo MX5 Microbalance (sensitivity: 0.01 mg) was used to measure the mass of electrode (substrate + WO_3_ nanostructures) and bare substrate and the mass loading of each sample is 2 mg/cm^2^.

### 2.3. Characterization

The morphological analyses were carried out by a scanning electron microscope (SEM) Gemini Field Emission SEM Carl Zeiss SUPRATM 25 (FEG-SEM, Carl Zeiss Microscopy GmbH, Jena, Germany) in In-LENS mode and by a transmission electron microscope (TEM) JEOL, JEM-ARM200F operated in TEM mode. Film structure was analyzed through XRD using a Smartlab Rigaku diffractometer, in grazing incidence 0.5°, equipped with a rotating anode of Cu Kα radiation operating at 45 kV and 200 mA. The scans were acquired from 10° to 70° with a step of 0.02°. The electrochemical measurements were performed at room temperature by using a potentiostat (VersaSTAT 4, Princeton Applied Research, Oak Ridge, TN, USA) and a three-electrode setup with a platinum counter electrode, a saturated calomel electrode (SCE) as reference, WO_3_ cast GP as working electrode (10 × 10 mm^2^ immersed area), in 1 M H_2_SO_4_ supporting electrolyte. Cyclic voltammetry (CV) curves were recorded at different scan rates (5 to 100 mV/s) in the −0.5 V to 0 V vs. SCE potential range and galvanostatic charge–discharge (GCD) tests were conducted at different current densities (0.5 to 5 A/g) in the potential range −0.5 ÷ 0 V.

## 3. Results and Discussion

### 3.1. WO_3_ Nanorods-Based Electrodes

**Figure 1:**
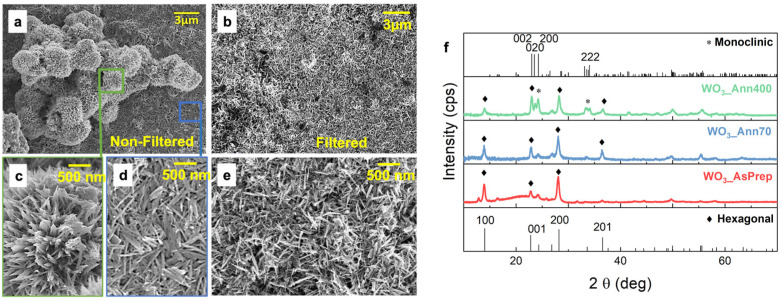
Low magnification SEM images of (**a**) NF and (**b**) F WO_3_ powder; high-resolution SEM images of (**c**) urchin-like nanostructures and (**d**) nanorods present in the NF WO_3_ powder and (**e**) nanorods present in the F WO_3_ powder; (**f**) XRD patterns of WO_3__AsPrep, WO_3__Ann70 and WO_3__Ann400 powders compared with the hexagonal and monoclinic characteristic patterns. The characteristic peaks are labeled depending on the crystalline planes, and hexagonal and monoclinic contributions are distinguished by using different symbols (rhombuses and circles, respectively).

WO_3_ powder appears as composed of large or small aggregates of nanorods if NF or F solution is used, respectively, as seen by low magnification SEM images in Figure 1a,b. The NF WO_3_ powder is characterized by urchin-like agglomerates (about 3 μm in size) of nanorods (Figure 1c), stacked on top of a thick WO_3_ nanorods film (Figure 1d). Instead, the F WO_3_ powder shown in Figure 1b does not have urchin-like agglomerates but only oddly aligned nanorods 0.5–1 μm long and 50 nm large, as high magnification SEM images in Figure 1e shows. It should be noted that the thermal treatment does not change the morphology of the WO_3_-based powder (Figure S1).

Figure 1f shows the XRD patterns of WO_3__AsPrep, WO_3__Ann70, and WO_3__Ann400 powders compared with XRD patterns of hexagonal (PDF #89-4476) and monoclinic (PDF #75-2187) WO_3_, respectively. The XRD pattern of the WO_3__AsPrep powders is not well defined, with little pronounced peaks at typical positions of hexagonal phase 2Ɵ = 14.00° for the plane (100), 24.36° for the plane (110), 26.84° for the plane (101), 28.22° for the plane (200), thus suggesting a poor crystalline nature of these powders, and a bump at around 2Ɵ = 20.00°, which can be associated with an earlier unknown nonstoichiometric phase [22]. Instead, well-defined typical diffraction peaks of the hexagonal structure are shown in the XRD pattern of WO_3__Ann70, which appear at 2Ɵ = 14.00°, 24.36°, 26.84°, 28.22°, 33.62°, 36.58°, and 49.95° thus confirming its pure hexagonal phase [21]. Moreover, peaks clearly related to the monoclinic crystal structure appear at 2Ɵ = 23.00°, 23.50°, 24.28°, 33.12°, 33.54°, 33.84°, 34.04°, 49.74°, 55.71° when the WO_3_ powder is annealed at 400 °C, by suggesting a partial phase transition and the coexistence of hexagonal and monoclinic WO_3_ phases (*h*-WO_3_ and *m*-WO_3_) in WO_3__Ann400 [23].

**Figure 2:**
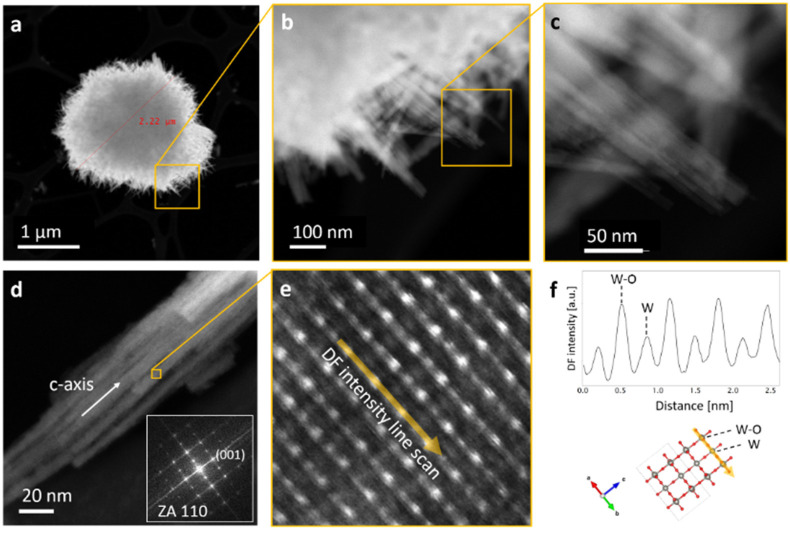
STEM Z-contrast micrographs of (**a**–**c**) a single urchin-like nanostructure at increasing magnification and (**d**) a single nanorod, composed of aligned nanoneedles; (**e**) enlarged view of the atomic lattice of a WO_3_ nanoneedle (**f**) DF intensity line scan of (**e**) and 3D atomic model of the WO_3_ hexagonal unit cell. Inset: FFT of (**d**)**.**

Scanning TEM analyses (STEM) were performed to investigate the morphology and crystal structure of single urchin-like nanostructure (Figure 2a–c) and nanorod (Figure 2d) from WO_3__AsPrep_NF powder. Figure 2a–c shows a single urchin-like structure and a portion thereof at different magnifications, thus revealing that it is composed of a bundle of nanorods. STEM image of Figure 2d reveals that each nanorod is composed of a bundle of very small needles with a section around 10 nm. These needles are well aligned with each other with the main dimension along the c-axis, as shown by the fast Fourier transform (FFT) on the inset of Figure 2d. The enlarged view of a WO_3_ nanoneedle in the high-resolution STEM image of Figure 2e confirms the hexagonal structure of the crystal lattice. In this figure, the brighter spots correspond to atomic columns with a bigger effective atomic mass (two tungsten and one oxygen atom per unit cell) while darker spots correspond to a lower effective atomic mass (atomic column with one tungsten atom per unit cell), as shown by the dark-field (DF) intensity line scan and by the 3D atomic model of WO_3_ hexagonal unit cell in Figure 2f. These results show that hydrothermal synthesis provides hexagonal WO_3_ needles stuck together to build nanorods and, hierarchically, urchin-like structures.

### 3.2. Evaluation of Energy Storage Performances

**Figure 3:**
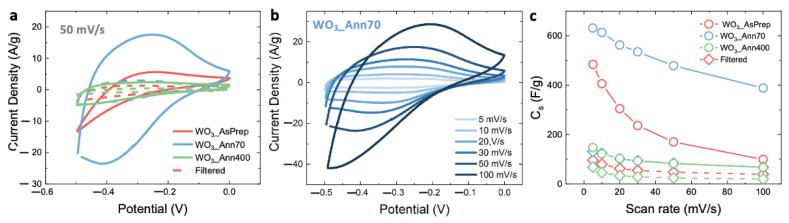
(**a**) CV curves obtained at 50 mV/s of NF (solid lines) and F (dotted lines) WO_3__AsPrep, WO_3__Ann70 and WO_3__Ann400 pastes electrodes; (**b**) CV curves of WO_3__Ann70 electrode obtained at different scan rate; (**c**) *C_s_* as a function of scan rate obtained from CV curves of NF (solid lines) and F (dotted lines) WO_3__AsPrep, WO_3__Ann70 and WO_3__Ann400 pastes electrodes. The potential is measured versus SCE.

The different morphologies and crystalline structures of WO_3_-based nanostructures lead to very diverse electrochemical performances, as shown below. Crystalline structure and morphology affect the energy storage performances, in terms of active storage mechanisms, and specific capacitance. To correlate the effects of crystalline structure and nanostructure typology to the energy storage mechanism, we prepared GP electrodes with NF and F WO_3_-based powder pastes, as described above. Figure 3a shows CV curves (from −0.5 V to 0 V at 50 mV/s) of electrodes obtained by using the NF (solid line) and F (dotted line) WO_3__AsPrep, WO_3__Ann70 and WO_3__Ann400 pastes-based electrodes (red, blue, and green lines, respectively). Electrodes obtained with filtered (F) pastes show similar quasi-rectangular CV curves, with a relatively small area under the CV curve. Larger areas are observed when using NF WO_3_ pastes. The largest CV curve is obtained for the WO_3__Ann70 powder, thus suggesting that WO_3_ with pure hexagonal crystal phase and urchin-like nanostructures and nanorods morphologies give the best performances for energy storage application.

Based on the above consideration, the NF WO_3__Ann70 electrode results are the most appropriate for investigating the energy storage mechanism. Figure 3b shows CV curves performed at different voltage scan rates (from 5 to 100 mV/s). CV curves of NF WO_3__AsPrep and WO_3__Ann400 electrodes, and of F WO_3__AsPrep, WO_3__Ann70, and WO_3__Ann400 electrodes are reported in Figure S2. All WO_3_-based electrodes show similar trend for CV curves, despite the area under the curve. No obvious redox peaks can be observed in the CV curves of Figure 3b and the curves exhibit the typical shape described for WO_3_-based electrodes [15,17,20,24,25,26]. CV curves significantly change by increasing the potential scan rate from 5 to 100 mV/s tending to a squarer shape. Indeed, the lower ion intercalation at higher scan rates could result in a weakening of the diffusion-controlled storage mechanisms, thus leading to charge storage driven by surface contributions. The coexistence of diffusion and surface-limited contributions results in a pseudocapacitive behavior, as expected [1,8,19,20]. Moreover, the absence of redox peaks in CV curves of Figure 3b and the quasi-rectangular shape indicate the presence of reversible and fast surface redox reactions at a constant rate. The specific capacitance (or pseudocapacitance) (*C_s_*) can be defined from CV curves as follows [6]:

(1) $$C_{s}=\frac{\int IdV}{m\upsilon ∆V}\;$$

where *I* is the measured current (mA), *V* is the measured potential (V), *m* is the active WO_3_ mass (mg), *υ* is the voltage scan rate (V/s) and ∆*V* is the potential range (V). Figure 3c shows the *C_s_* value as a function of the voltage scan rate of NF (circled symbols) and F (rhombohedral symbols) WO_3__AsPrep, WO_3__Ann70 and WO_3__Ann400 pastes-based electrodes (red, blue, and green symbols, respectively). The highest *C_s_* values are obtained for the NF WO_3__Ann70 (632 F/g at 5 mV/s), as expected, while the NF WO_3__AsPrep and WO_3__Ann400 paste-based electrodes show low *C_s_*, by confirming the poor energy storage activity of WO_3_ nanostructures with poor crystalline nature or with a mixture of crystalline phases. The high *C_s_* value obtained for the WO_3__Ann70 electrode can be ascribed to the high crystalline nature and the pure hexagonal structure, which presents favorable H^+^ intercalation–release pathways [20]. It should be emphasized that as-prepared WO_3_ nanorods, even if with hexagonal peaks in the XRD spectrum, do not give the best results, and a soft annealing up to 70 °C is needed to greatly improve the storage efficiency. On the other hand, strong calcination (as at 400 °C) is detrimental because the hexagonal phase starts to be lost. Additionally, the presence of urchin-like nanostructures affects the *C_s_* since this morphology contributes to increasing the porosity of the electrochemical active film. Such porosity may improve the mass transport of electrolyte ions to the entire electrochemical active surface, useful when high power densities are required because of the improvement of available electrochemical active surface [1]. The *C_s_* of electrodes obtained from F WO_3_ pastes is very low compared to those obtained from NF WO_3_ pastes. This is probably due to the low porosity of the dense film of WO_3_ nanorods, as shown in SEM images of Figure 1b,e. 

All the electrodes show the same trend of *C_s_* as a function of scan rate: as the scan rate increases, the *C_s_* decreases. Such a feature of *C_s_* confirms the above assumptions, for which diffusion and surface-controlled storage mechanisms act at the same time depending on the voltage scan rate: at a lower scan rate, both mechanisms are active which results in higher *C_s_*, while at a higher scan rate only surface mechanisms are actively inducing a *C_s_* decrease. 

**Figure 4:**
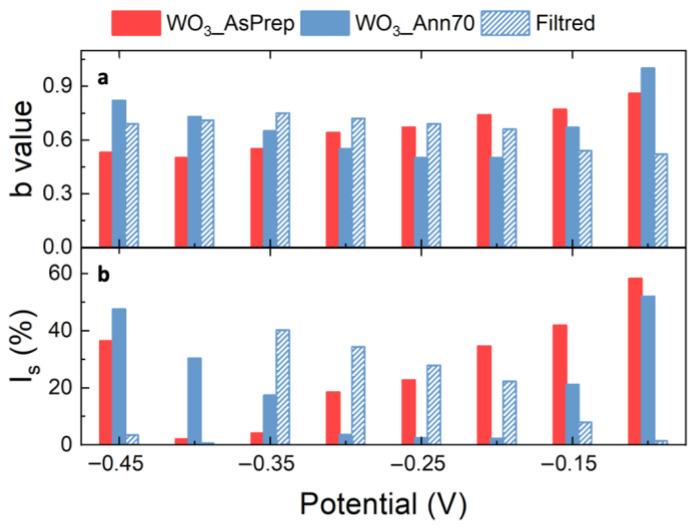
Comparison of (**a**) b values and (**b**) of surface-limited current contributions (I_s_) obtained for NF WO_3__AsPrep and WO_3__Ann70 and F WO_3__Ann70 pastes electrodes and as a function of applied potential at 5 mV/s. The potential is measured versus SCE.

To study the role of crystalline quality and morphology of WO_3_ nanostructures in energy storage activity, the CV curves were deeply analyzed and described in terms of surface and diffusion-limited contribution. Generally, at fixed potential *V* the current (*i*) depends on scan rate (υ) as follows [20]:

(2) $$i\left(V\right)=a\upsilon^{b}$$

where *a* and *b* are parameters. At the electrode/electrolyte interface, if the charge exchange is limited by diffusion of ions (as in an ideal battery), *b* is equal to 0.5, while if the charge exchange is limited by surface process (as in ideal EDLC), then *b* is equal to 1 [10,20]. By considering the CV curves of NF WO_3__AsPrep and WO_3__Ann70 and F WO_3__AsPrep pastes-based electrodes performed at 5, 10, 20, and 30 mV/s, the cathodic current values at fixed potentials are taken into consideration for the determination of *b* value. From the linear fits of ln(*i*) as a function of ln(υ), shown in Figure S3a–c, *b* value is extracted and reported as a function of potential in Figure 4a,b. Figure 4a shows the comparison between the obtained *b* values as a function of potential for NF WO_3__AsPrep and WO_3__Ann70 and for F WO_3__Ann70 electrodes (full red, full blue, and with blue pattern column, respectively), to highlight the role of crystalline structure and morphology in energy storage. The *b* value ranges between 0.5 and 1, thus confirming the pseudocapacitor behavior of tested WO_3_-based electrodes. The pure hexagonal crystalline structure of the WO_3__Ann70 electrode (full blue column) contributes to improving the surface role at more negative potential and at potential close to 0 V (*b* tends to 1), while the diffusion contributions are well present at around −0.2 to −0.1 V (*b* tends to 0.5). This results in a clear potential region separation for the activation of surface and diffusion-limited processes. The low crystallinity of the WO_3__AsPrep electrode (full red column) leads to a mixture of surface and diffusion contribution to almost all intermediate potentials, with a *b* value that gradually switches from 0.5 at −0.5 V to 1 at 0 V. Figure 4a describes also the role of WO_3_ morphology in the energy storage activity by comparing *b* values obtained from CV curves of NF and F WO_3__Ann70 paste-based electrodes (full blue and with blue pattern column, respectively). The absence of urchin-like nanostructures in F pastes leads to a lowering of surface-limited contributions at all potentials (*b* value always lower than 0.8). Taking into account the above consideration, the better energy storage performances of NF WO_3__Ann70 can be ascribed to the activation of surface-limited contributions, which seem to be more effective than the diffusion-limited ones for almost all potentials. The surface-limited contributions are mainly given by the presence of hexagonal urchin-like nanostructures, which, thanks to their morphology, possess a larger surface area immersed in the electrolyte solution.

The kinetics of electrochemical processes can be studied by applying the model proposed by Dunn et al. [10], which allows the determination of diffusion and surface-limited current contributions to the measured total current during CV analysis. According to this model, the total current at fixed potential *i*(*V*) depends on scan rate υ as follows:

(3) $$i\left(V\right)=k_{1}\upsilon +k_{2}\upsilon^{1/2}$$

where $k_{1}$ and $k_{2}$ are the coefficients related to surface and diffusion contributions to the total current, respectively. The kinetics analysis described above is applied for low scan rates, to avoid errors due to the polarization and to the ohmic losses occurring at high scan rates [20,27].

**Figure 5:**
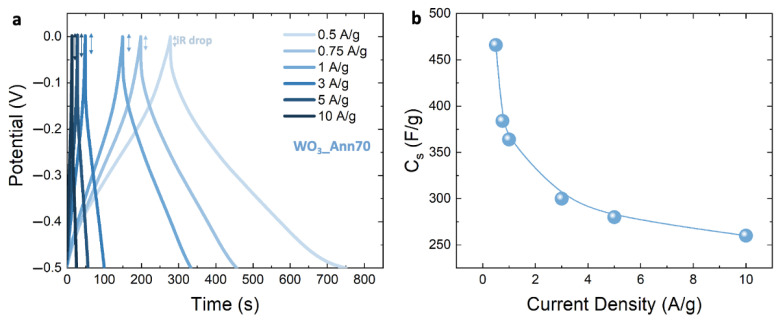
(**a**) GCD curves of NF WO_3__Ann70 paste-based electrode obtained in 1 M H_2_SO_4_ solution at different current densities. The iR drop is indicated for each GCD curve; (**b**) *C_s_* as a function of current density obtained from the GCD curves; potential measured versus SCE.

Figure S3d–f shows the $I\left(V\right)\upsilon^{-1/2}$ plot as a function of $\upsilon^{1/2}$ obtained from CV curves of NF WO_3__AsPrep and WO_3__Ann70 and the F WO_3__AsPrep electrodes performed at 5, 10, 20 and 30 mV/s. $k_{1}$ and $k_{2}$, and consequently, the diffusion and current contributions can be defined from the linear fits (details of calculation in Supplementary Material). Figure 4b shows the comparison between surface-limited current contributions (*I_s_*) calculated at 5 mV/s as a function of potential for NF WO_3__AsPrep and WO_3__Ann70 and for F WO_3__Ann70 electrodes (full red, full blue, and with blue pattern column, respectively). *I_s_* increases as *b* approaches 1, as expected, thus confirming that the better energy storage performances of NF WO_3__Ann70 paste electrodes are strictly correlated to the presence of diffusion-limited mechanisms. The diffusion-limited current contribution (*I_d_*) is reported in Figure S4. CV is a powerful tool for electrochemical behavior comprehension, since CV analyses allow us to define the suitable potential range for electrochemical characterization and the type of electrochemical reactions (diffusion or surface limited). Unfortunately, *C_s_* calculated from CV curves represent an approximation of the real value, and more in-depth and robust electrochemical analysis is required to unveil energy storage activity in terms of power and energy densities, and series resistance [28]. The characterization of the charge–discharge behavior through GCD analysis is the most widely used method [1,3,10,28]. GCD curves of NF WO_3__Ann70 paste electrode are studied, to consolidate its energy storage activity. Figure 5a shows the GCD curves obtained at different current densities (from 0.5 to 10 A/g) in the voltage range from −0.5 to 0 V: the discharge time of the hexagonal WO_3_ nanostructures significantly decreases as the current density increases, as expected. The charge and discharge curves are symmetric, indicating poor energy loss, high reversibility, and high columbic efficiency during the charge and discharge process [7,29]. Moreover, the GCD curves display a non-linearity at different current densities, thus suggesting that surface and diffusion-limited processes occur at the interface between electrolyte and electrode [15]. Moreover, the GCD curves show an IR drop related to the internal resistance of the electrode (see details in Figure 5a) which increases as the current density increases, thus suggesting that at higher current density fewer active sites are accessible for the electrochemical reaction [25]. The resulting equivalent series resistance is 30, 28, 27, 23, 20, and 16 Ω at current densities of 0.5, 0.75, 1, 3, 5, and 10 A/g. *C_s_* can be calculated also from the GCD curve as follows [8]:

(4) $$C_{s}=\frac{It_{s}}{m∆V}$$

where *I* is the applied current (mA), *t_s_* is the discharge time, *m* is the active WO_3_ mass (mg), and ∆V is the potential range (V). Figure 5b shows the related *C_s_*: as the current density increases, the *C_s_* decreases from 466 F/g at 0.5 A/g, to 355 F/g at 1 A/g, to 260 F/g at 5 A/g, which are slightly lower than the results previously obtained from CV curves because of the chosen current density [28], but in accordance with them. The sharp decreases in the *C_s_* values can be associated with the quenching of the diffusion-limited contributions at high current density, further confirming the above assumption [30].

### 3.3. Asymmetric Supercapacitor Realization

To evaluate the energy storage activity of WO_3_-based electrodes in realistic conditions, an asymmetric supercapacitor (ASC) has been realized, in which the above-characterized electrode and a GP electrode act as anode and cathode, respectively, in a two-electrode setup with 1 M H_2_SO_4_ electrolyte solution. The mass ratio between the anode (WO_3_-based electrode) and the cathode (GP substrate) is calculated using the charge balance theory (Q_+_ = Q_−_) as follows [31,32,33]:

(5) $$\frac{m_{-}\;}{m_{+}}=\frac{C_{s}{}_{+}\times ∆V_{+}\;}{C_{s}{}_{-}\times ∆V_{-}}\;$$

In which *m_−_*, *m_+_, C_S−_*, *C_S+_,* ∆*V_−_*, and ∆*V_+_* are the mass of the electrochemically active material, the specific capacitance obtained from CV analysis, and the potential interval in which the electrochemical tests are performed of the anode and of the cathode, respectively, (the comparison between the CV profile is reported in Figure S5, as well as the CV and the GCD curves of the GP acquired at different scan rates and currents value, respectively).

**Figure 6:**
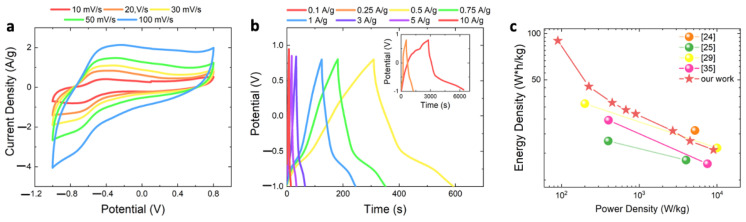
(**a**) CV curves at different scan rates of the ASC realized by using GP as anode and NF WO_3__ann70 paste electrode (schematic illustration in the inset); (**b**) GCD of ASC in the potential range between −1 to 0.8 V. The inset shows a magnification of GCD curves at higher current density; (**c**) Ragone plot obtained from the as tested WO_3_-based ASC (red stars) compared to those reported in the literature for WO_3_-based ASC. Potential is measured versus SCE.

By using an asymmetric configuration, a wide potential range can be explored to reach the maximum value of energy and power density [34]. To evaluate the *C_s_*, CV and GCD were performed in the operating voltage range from −1 V to 0.8 V. Figure 6a shows CV curves obtained at different scan rates (from 5 to 100 mV/s). The proportional expansion of CV curves as the scan rate increases reveals a fast charging/discharging capability [19].

At negative potentials, the CV curves are quite similar to those of the single WO_3_-based electrode (see Figure 3b for more details). Figure S6 a shows the *C_s_* obtained from CV curves as a function of the scan rate: the *C_s_* decreases as the scan rate increases from 232 F/g at 10 mV/s to 115 F/g at 100 mV/s. The relatively high values of *C_s_* confirm the promising charge storage characteristics of WO_3_. This can probably be ascribed to the high wettability of the WO_3_ urchin-like nanostructures (see details in the high magnification SEM image in Figure 1c), which is useful for increasing the electrochemically active surface.

Figure 6b shows the GCD curves obtained at different current densities (from 0.5 A/g to 3 A/g, the highest and lowest current density curves are reported in Figure S7) in the potential ranges from −1 V to 0.8 V. The charge and discharge curves are asymmetric, again confirming the existence of a pseudocapacitive behavior of the WO_3_-based electrode [29]. The *C_s_* as a function of the current density is reported in Figure S5b. As the current density increases, the *C_s_* decreases from 200 F/g at 0.1 A/g to 40 F/g at 10 A/g, as expected.

Energy and power density (*E_d_* (W × h/kg) and *P_d_* (W/kg), respectively) are the most important indexes used to compare energy storage devices, and can be defined from GCD analysis as follows [19]:

(6) $$E_{d}=\frac{1}{2\times 3.6}C_{s}∆V^{2}$$

(7) $$P_{d}=E_{d}\times 3600/∆t$$

where $C_{s}$ is the specific capacitance obtained from GCD curves, ∆V is the potential interval (1.8 V), ∆t is the discharge time and the coefficients 3.6 and 3600 are related to the conversion of the measurement units (from s and g to h and kg, respectively). The as-tested ASC device shows the highest *E_d_* of 90 W × h/kg at *P_d_* of 90 W/kg and the highest *P_d_* of 9000 W/kg at *E_d_* of 18 W × h/kg (see all the details in the Supplementary Material, Table S1). Figure 6c shows the Ragone plot (*P_d_* reported as a function of *E_d_*)_._ Values related to our WO_3_-based ASC are reported as red stars, for each analyzed current density, while the spheres are data reported from the literature (see color legend for references). In a Ragone plot, batteries lie in the bottom right corner, while capacitors stay in the upper left corner; pseudocapacitors bridge the gap. Our data span a very wide range of performances with many experimental points, which is a key feature for the maximization of *E_d_* and *P_d_*. The *E_d_* and *P_d_* obtained in the present case are higher than those of several previously reported ASC based on WO_3_-WO_3_∙0.5H_2_O mixtures (23.4 W × h/kg at 5200 W/kg, orange ball) [24], RuO_2_/h-WO_3_ nanorods (20 W × h/kg at 400 W/kg and 15 W × h/kg at 4000 W/kg green balls) [25], carbon–WO_3_ nanocomposite (35 W × h/kg at 200 W/kg and 18 W × h/Kg at 10,000 W/kg, yellow balls) [29], WO_3_ nanorods bundles (27 W × h/kg at 403 W/Kg and 14 W × h/kg at 7529 W/kg, violet balls) [35]. These promising results can be ascribed to the good agreement between the highly pure hexagonal crystal structure and the urchin-like morphology, which allows us to increase the electrolyte exposed surface, as well as the wettability of WO_3_-based electrodes.

## 4. Conclusions

In conclusion, WO_3_ nanostructures, synthesized by simple and low-cost hydrothermal technique, followed by air calcination, were investigated as potential candidates for electrodes in pseudocapacitors. The WO_3_ nanostructures crystal structure strongly depends on the annealing temperatures, reaching a pure hexagonal structure after air annealing at 70 °C. As synthesized WO_3_ nanostructures present two typologies (3 µm large urchin-like and much smaller nanorods) which have been selected by the filtering procedure. TEM analysis unveiled 10 nm thin nanoneedles as the fundamental building block for both urchin-like and nanorods typologies. The electrochemical energy storage mechanisms are described in terms of surface and diffusion-limited processes, highlighting the role of morphology and crystal structure. Exceptional energy storage activity is obtained for the pure hexagonal WO_3_ nanostructures as long as both urchin-like nanostructures and nanorods are present, with a *C_s_* of 632 F/g at 5 mV/s and of 466 F/g at 0.5 A/g. This electrode was used for the realization of an asymmetric supercapacitor, showing promising performances over a large range of energy and power densities.

## Data Availability

The data presented in this study are available on request from the corresponding author.

## Supplementary Materials

The following supporting information can be downloaded at: https://www.mdpi.com/article/10.3390/nano12234168/s1: Figure S1: High and low magnification SEM images of (a,d) WO_3__AsPrep, (b,e) WO_3__Ann70 and (c,f) WO_3__Ann400 pastes, respectively; Figure S2: CV curves at different scan rates of NF and F (a,b) WO_3__AsPrep, (c,d) WO_3__Ann400 and (e) WO_3__Ann70 pastes, respectively. Figure S3: plots of ln(*I*) as a function of ln(*v*) at different potential values obtained by considering CV curves of (a) NF WO_3__AsPrep, (b) F WO_3__Ann70 and (c) WO_3__Ann70 pastes-based electrodes at 5, 10, 20 and 30 mV/s at fixed potentials. The b value can be determined from the linear fit of curves obtained at fixed potential; plot of *I*(*V*) *υ*^−1/2^ as a function of *υ*^−1/2^ for the determination of surface and diffusion contributes to the total current measured during CV analysis of at (d) NF WO_3__AsPrep, (e) F WO_3__Ann70 and (f) WO_3__Ann70 pastes-based electrodes at 5, 10, 20 and 30 mV/s at fixed potentials. The linear fits allows to determine K_1_ and K_2_ at each potential. Figure S4: Comparison of diffusion-limited current contribution (Id) for (a) NF WO_3__AsPrep and WO_3__Ann70 pastes-based electrodes and (b) NF and F WO_3__Ann70 pastes-based electrodes as a function of the applied potential. The potential is measured versus SCE. Figure S5: Comparison between CV profiles acquired at 5 mV/s of the GP and of the WO_3__Ann70 electrodes; (b) CV curves acquired at different scan rate and (c) GCD curves acquired under different current values of GP electrode. Figure S6: *C_s_* as a function of (a) scan rate and (b) current density obtained from the GCD curves of the WO_3_-based ASC. Figure S7: GCD of the asymmetric supercapacitor obtained at (a) lower and (b) high current density. Table S1: Electrochemical parameters obtained for the ASC device.
