# Supplementary material for: Engineering of Nanostructured WO3 Powders for Asymmetric Supercapacitors
Source: Nanomaterials (Basel). 2022 Nov 24;12(23):4168. doi: 10.3390/nano12234168 (PMC9738873; doi:10.3390/nano12234168)
Supplement: Supplementary file 1 [file nanomaterials-12-04168-s001.zip › nanomaterials-2011765-supplementary/nanomaterials-2011765-supplementary materials.pdf]

# Engineering of nanostructured $WO_3$ powders for asymmetric supercapacitors

**Giacometta Mineo [1,2], Mario Scuderi [3], Gianni Pezzotti Escobar [2], Salvo Mirabella [1,2,*] and Elena Bruno [1,2]**

[1] Physics and Astronomy Department"Ettore Majorana", University of Study of Catania, via S. Sofia 64, 95123 Catania, Italy
[2] CNR-IMM, Catania (University) Unit, via S. Sofia 64, 95123 Catania, Italy; gianni.pezzotti@imm.cnr.it
[3] CNR-IMM VIII strada 5, 95121, Catania, Italy
* Correspondence: salvo.mirabella@dfa.unict.it

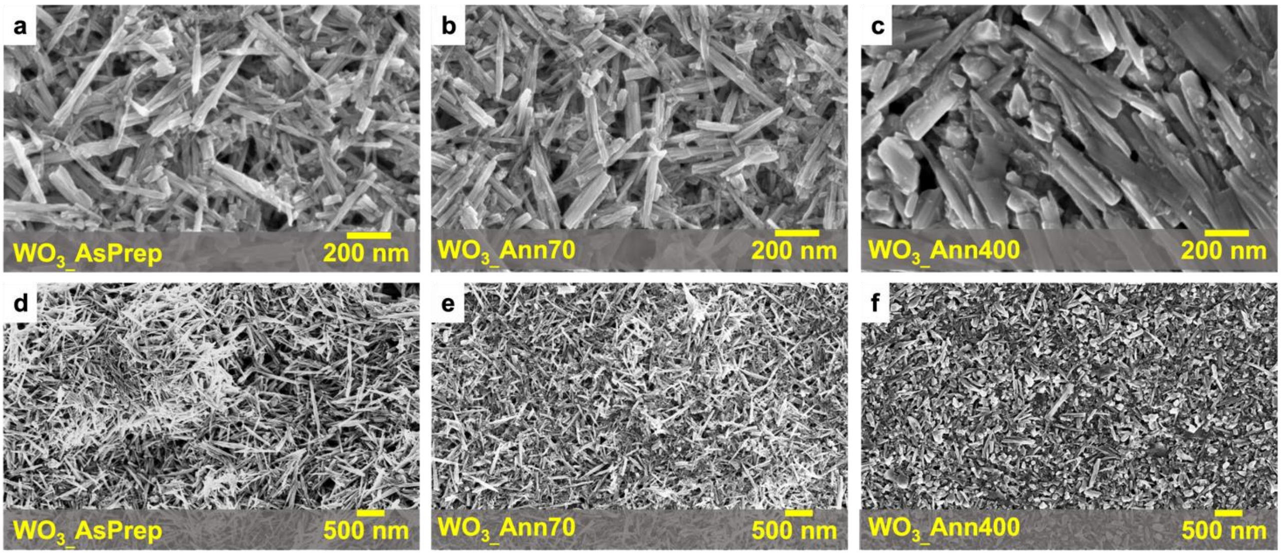


**Figure S1.** High and low magnification SEM images of (a and d) $WO_3$_AsPrep, (b and e) $WO_3$_Ann70 and (c and f) $WO_3$_Ann400 pastes respectively.

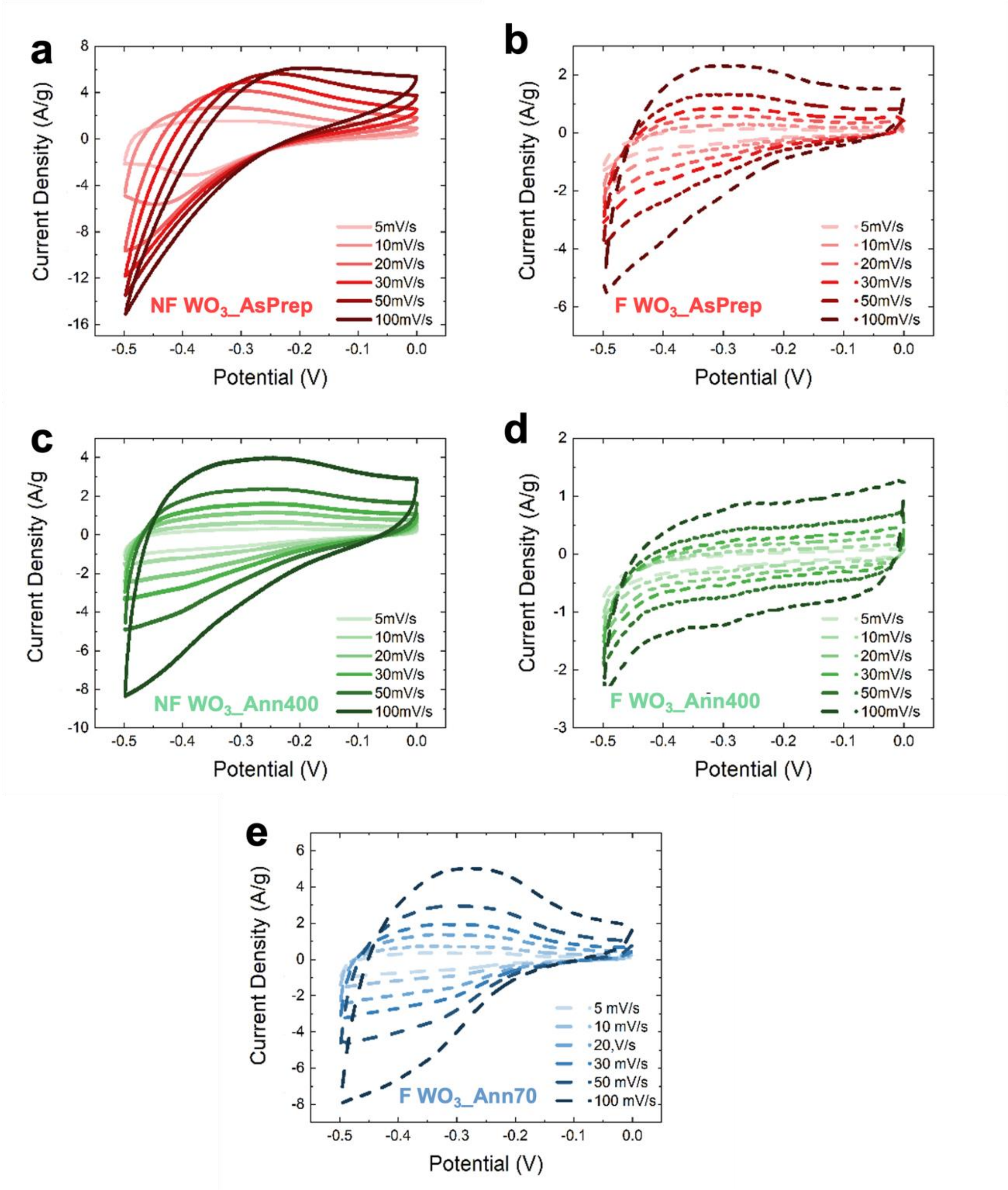


**Figure S2.** CV curves at different scan rates of NF and F (a and b) $WO_3$_AsPrep, (c and d) $WO_3$_Ann400 and (e) $WO_3$_Ann70 pastes, respectively.

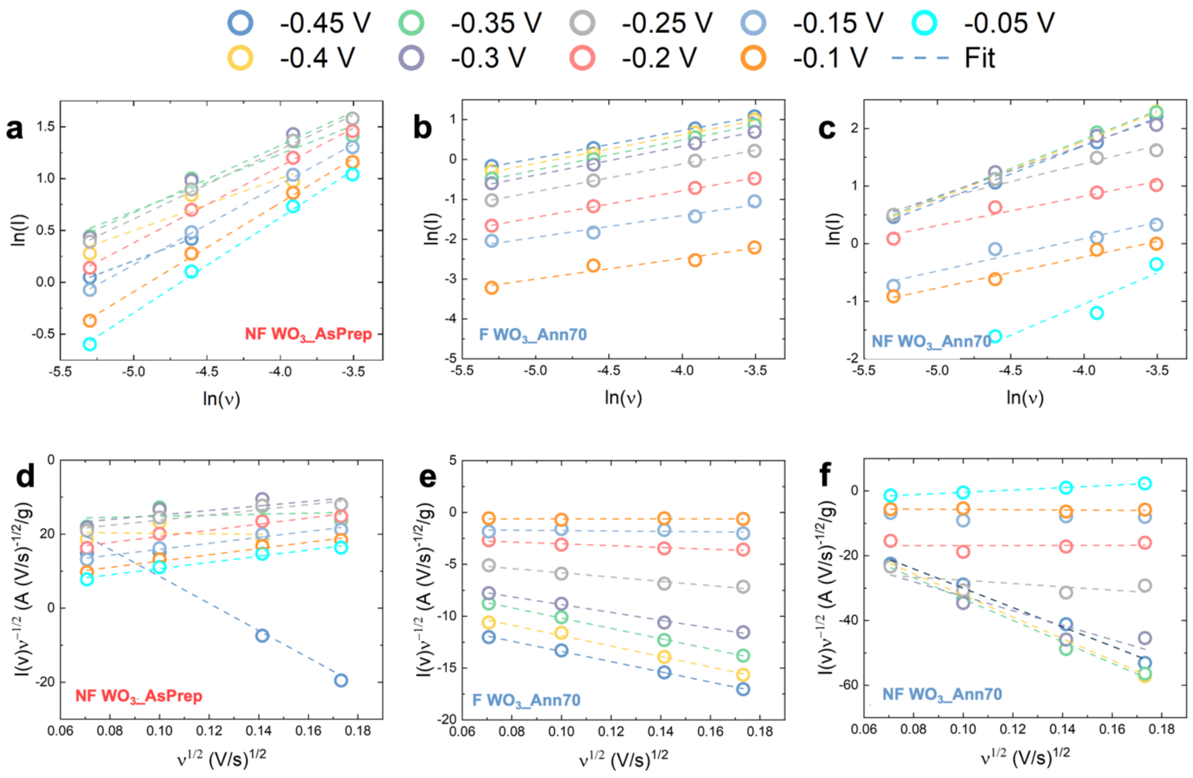


**Figure S3.** plots of ln(I) as a function of ln(v) at different potential values obtained by considering CV curves of (a) NF $WO_3$_AsPrep, (b) F $WO_3$_Ann70 and (c) $WO_3$_Ann70 pastes-based electrodes at 5, 10, 20 and 30 mV/s at fixed potentials. The b value can be determined from the linear fit of curves obtained at fixed potential; plot of $I(V)\upsilon^{-1/2}$ as a function of $\upsilon^{1/2}$ for the determination of surface and diffusion contributes to the total current measured during CV analysis of at(d) NF $WO_3$_AsPrep, (e) F $WO_3$_Ann70 and (f) $WO_3$_Ann70 pastes-based electrodes at 5, 10, 20 and 30 mV/s at fixed potentials. The linear fits allows to determine $K_1$ and $K_2$ at each potential

***Calculation of current contribution***

The equation:

$$i(V) = k_1 \upsilon + k_2 \upsilon^{1/2}$$

Can be rewriting as a follow:

$$i(V)\upsilon^{-1/2} = k_1 \upsilon^{1/2} + k_2$$

From the linear fit of $i(V)\upsilon^{-1/2}$ as a function of $\upsilon^{1/2}$ the surface (*$k_1$*) and diffusion (*$k_2$*) contribution can be defined.

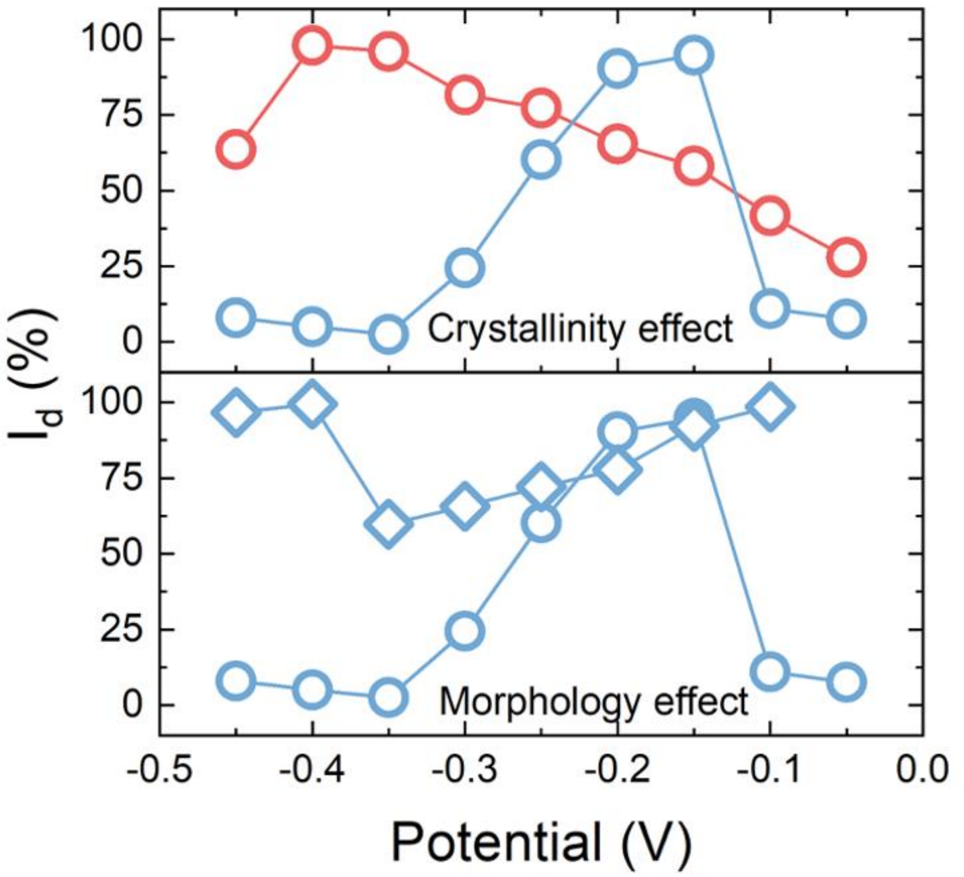


**Figure S4.** Comparison of diffusion-limited current contribution ($I_d$) for (a) NF $WO_3$_AsPrep and $WO_3$_Ann70 pastes-based electrodes and (b) NF and F $WO_3$_Ann70 pastes-based electrodes as a function of the applied potential. The potential is measured versus SCE.

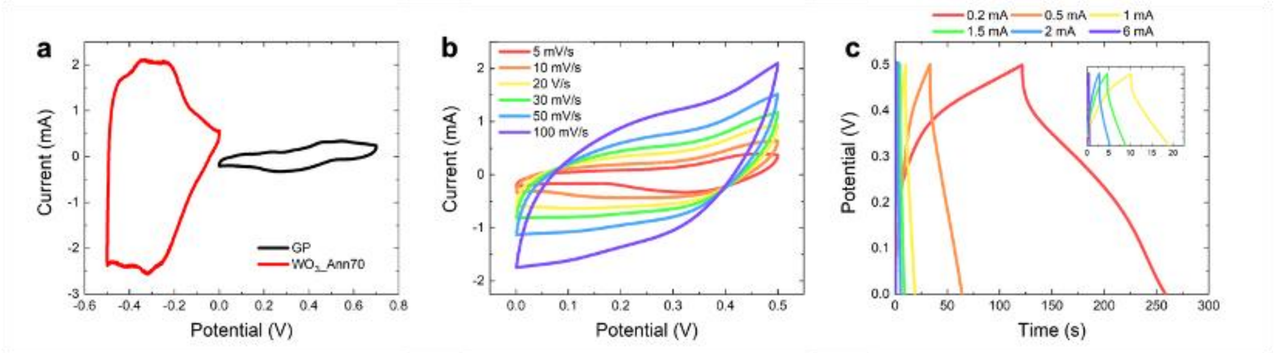


**Figure S5.** Comparison between CV profiles acquired at 5 mV/s of the GP and of the $WO_3$_Ann70 electrodes; (b) CV curves acquired at different scan rate and (c) GCD curves acquired under different current values of GP electrode.

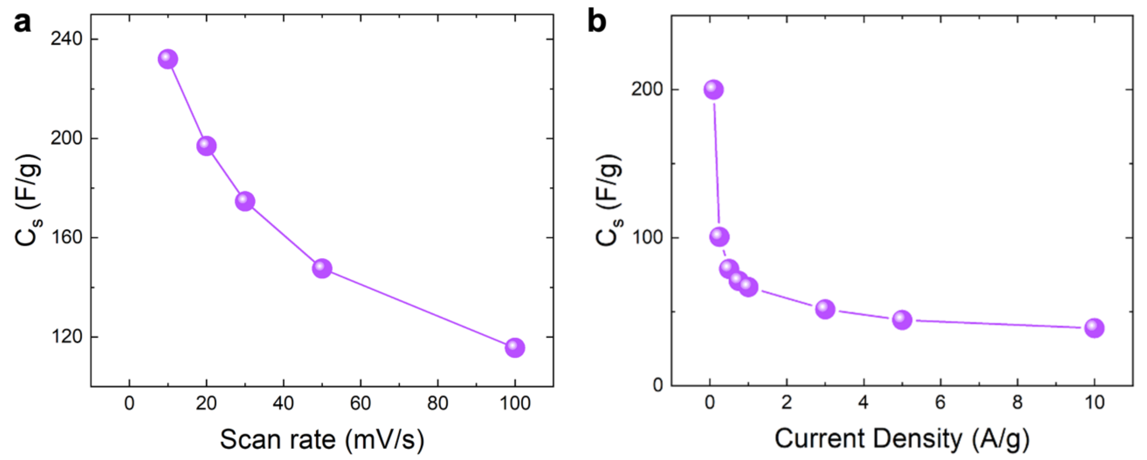


**Figure S6.** $C_s$ as a function of (a) scan rate and (b)current density obtained from the GCD curves of the $WO_3$ based ASC.

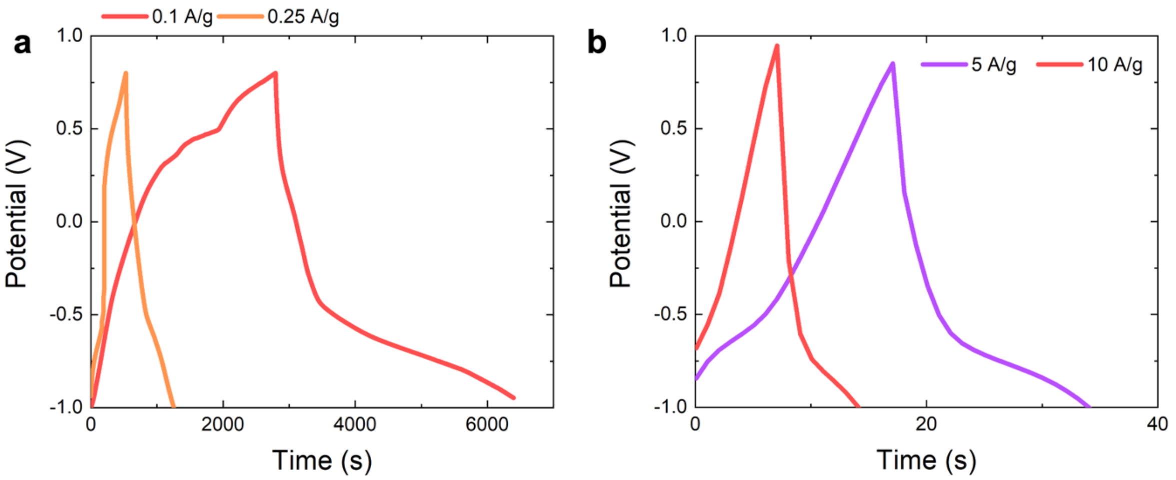


**Figure S7.** GCD of the asymmetric supercapacitor obtained at (a) lower and (b) high current density.

**Table S1.** Electrochemical parameters obtained for the ASC device.

| Current Density (A/g) | Discharge time (s) | Specific Capacitance (F/g) | Energy Density (W*h/kg) | Power Density (W/kg) |
|---|---|---|---|---|
| 0.1 | 3599 | 199.94 | 89.98 | 90 |
| 0.25 | 724 | 100.56 | 45.25 | 225 |
| 0.5 | 284 | 78.89 | 35.5 | 450 |
| 0.75 | 170 | 70.83 | 31.88 | 675 |
| 1 | 120 | 66.67 | 30 | 900 |
| 3 | 31 | 51.67 | 23.25 | 2700 |
| 5 | 16 | 44.44 | 20 | 4500 |
| 10 | 7 | 38.89 | 17.5 | 9000 |
